# Validation of the first computerized indicator for orthopaedic surgical site infections in France: ISO-ORTHO

**DOI:** 10.1186/s13756-023-01239-7

**Published:** 2023-05-04

**Authors:** Leslie Grammatico-Guillon, Linda Banaei-Bouchareb, Agnès Solomiac, Katiuska Miliani, Pascal Astagneau, Laetitia May-Michelangeli

**Affiliations:** 1 Service of Public Health, Epidemiology and data center, Teaching hospital of Tours and Medical School of Tours, Tours, France; 2 Medical School, University of tours, Tours, France; 3 Center for Prevention of Healthcare Associated Infection, INSERM, Institute of Epidemiology and Public Health, Sorbonne University, Paris, F75013 France; 4 French National Authority for Health (“Haute Autorité de Santé”, HAS), Saint Denis, France

**Keywords:** Surgical site infection, Total knee/hip arthroplasty, Hospital discharge database, Quality of healthcare, Surveillance, Methods

## Abstract

**Background:**

The French national authority for health (HAS) develops in-hospital indicators for improving quality of care, safety and patient outcome. Since 2017, it has developed a measurement of surgical site infections (SSI) after total hip or knee arthroplasty (TH/KA) by using a computerized indicator, called ISO-ORTHO, based on a hospital discharge database (HDD) algorithm. The aim of the study was to assess the performance of this new indicator .

**Methods:**

The ISO-ORTHO performance was estimated via its positive predictive value (PPV) among adult patients having undergone a TH/KA between January 1st and September 30th 2018, based on the orthopaedic procedure codes. Patients at very high risk of SSI and/or with SSI not related to the in-hospital care were excluded. SSI were detected from the date of admission up to 90 days after the TH/KA using the ISO-ORTHO algorithm, based on 15 combinations of ICD-10 and procedure codes. Its PPV was estimated by a chart review in volunteer healthcare organisations (HCO).

**Results:**

Over the study period, 777 HCO including 143,227 TH/KA stays were selected, providing 1,279 SSI according to the ISO-ORTHO indicator. The 90-day SSI rate was 0.89 per 100 TH/KA stays (0.98% for THA and 0.80% for TKA). Among the 448 HCO with at least 1 SSI, 250 HCO participated in reviewing 725 SSI charts; 665 were confirmed, giving a PPV of 90.3% [88.2-92.5%], 89.9% [87.1-92.8%] in THA and 90.9% [87.7-94.2%] in TKA.

**Conclusions:**

The PPV of ISO-ORTHO over 90% confirms its validity for any use according to the HAS method. ISO-ORTHO and detailed information were provided in 2020 to HCO and used for quality assessment and in-hospital risk management.

**Supplementary Information:**

The online version contains supplementary material available at 10.1186/s13756-023-01239-7.

## Introduction

Over the last few decades, the total number of hip (THA) and knee (TKA) arthroplasties performed annually in French hospitals has consistently increased reaching over 150,000 (French hospital discharge data 2017–2019, PMSI ATIH) [1–3]. This activity is rising due to the ageing population along with the increasing demands of patients in terms of functional capacities [4, 5]. Approximately 14.4% of patients report an adverse event during their surgical care, of which 5.2% are considered potentially preventable, especially after a joint replacement [6, 7]. The most frequent complications after THA and TKA (TH/KA) are infection with 1% of these surgeries subsequently resulting in surgical site infections (SSI) [8–11]. Due to the increasing frequency of arthroplasties and the potential impact of complications in terms of loss of quality of life and additional costs for the society [12, 13], SSI represent a key target for healthcare-associated infection surveillance [12–16].

In France, the SSI surveillance is one of the priority targets of the national program for the prevention of healthcare associated infections (*PROgramme national de Prévention des Infections Associées aux Soins*, PROPIAS [17]) as recommended by the High Council of Public Health. Whereas several countries are using national TH/KA registers for surveillance [18–23], no such dynamic and exhaustive national register exists in France. The French National Authority for Health (“Haute Autorité de Santé”, HAS) developed a national computerized SSI indicator in orthopaedic surgery for quality and safety improvement: ISO-ORTHO [27]. This indicator derived from an innovative French research project for SSI monitoring in orthopaedics, that validated an algorithm based on hospital discharge database (HDD) with an acceptable positive predictive value (PPV) of 87% [24–26]. In 2017, the HAS developed a revised version of this research SSI algorithm with a multidisciplinary working group composed of patients and experts of orthopaedics, anaesthesiology, epidemiology, infection control, medical information, and infectious diseases [27]. Subsequently, the HAS published the different methodological steps for its development and validation [19, 27–33]. Eventually, the definition of the ISO-ORTHO indicator was achieved through the optimization of SSI detection based on the coding practice guidelines of the Technical Agency for Information on Hospital Care (“Agence technique de l’information sur l’hospitalisation”, *ATIH*), along with redefining the exclusion criteria of the target population [34–36].

The aim of the present study was to validate the outcome indicator ISO-ORTHO by measuring its performance to detect SSI over the 90 days following a total TH/KA in France.

## Methods

### Study design and population

**Fig. 1:**
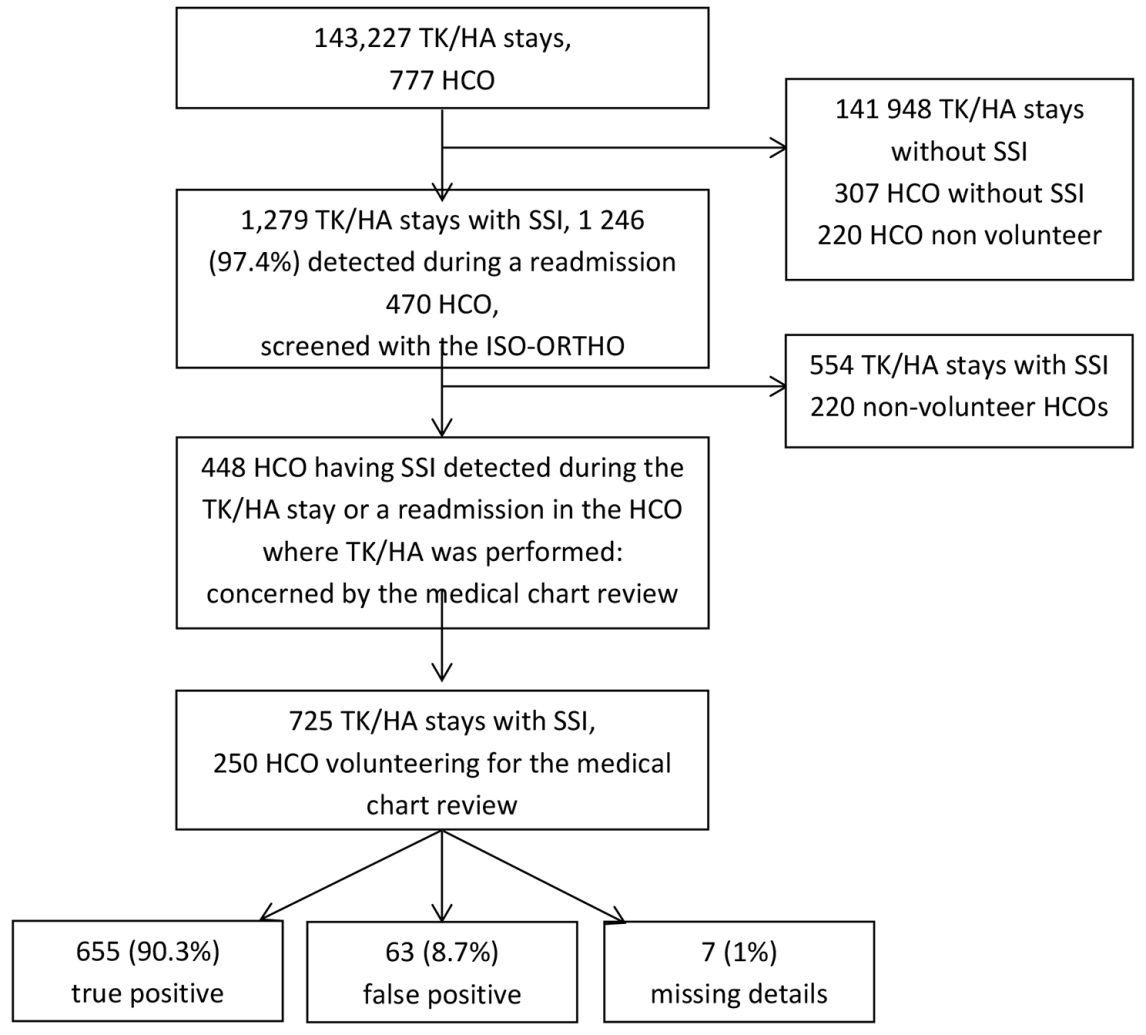
Flow chart of the reviewed medical chart (n = 725) *SSI Surgical site procedure; TK/HA Total knee hip arthroplasty; HCO Healthcare Organisation*

An evaluative study was performed to assess the performance of the ISO-ORTHO indicator via its PPV using a medical chart review. All adult patients with a hospital stay for TH/KA that occurred between January, 1st and September, 30th 2018 in the French national hospital discharge database were selected according to their surgical procedure for hip or knee replacement based on the French Common Classification of Medical Acts (THA: *NEKA010, NEKA012, NEKA013, NEKA014, NEKA015, NEKA016, NEKA017, NEKA019, NEKA020, NEKA021;* TKA: *NFKA007, NFKA008, NFKA009*). In case of multiple TH/KA stays, only the first was included. Exclusion criteria were patients at very high risk of SSI (history of complex SSI such as complex surgical procedures, another surgical act performed on hip or knee during the TH/KA stay, hip or knee surgery in the previous three months before hospital stay, hip fracture), with SSI not related to in-hospital care and/or data or linkage problems (error in the sex, birth date and/or social insurance number) (See Fig. 1, and arguments for inclusion and exclusion criteria detailed in *Appendix*A1).

**Table:** Footnote of Fig. 1. Flowchart of the target population, SSI and HCOs

Inclusion criteria	2018
Stays with at least 1 TH/TKA stay	158 478
**HCOs**	**791**
**Exclusion criteria**	
errors in the coding, misclassification, or linkage problems	730
age under 18	69
**Stays of adult patients with at least 1 TH/KA coded and without data and/or linkage problems**	**157 682**
admission for infection	117
infection not related to the THA or TKA procedure :	47
fracture as the reason for hip replacement	10 386
emergency admission	8 080
several acts of orthopaedic surgery in the same initial stay, or act of device change	677
mechanic complication coded during the stay	1 447
patients transferred from another HCO	1 424
patients with a hospitalisation between TH/KA and the readmission for SSI	106
hip or knee surgery in the previous three months before hospital stay	1 160
palliative care detected 1 year before, within or 3 months after the TH/KA stay	357
history of complex SSI detected in the previous year	87
patient coming from another country (non-French residents)séjours de patients résidant hors France (codes géographiques entre 99,101 et 99,517 + 99,999)	276
death during the surgical stay without any code of SSI	283
length of stay over 90 days	23
patients discharged against medical advice or escaped	46
**Excluded stays : N (%)**	**13 635 (9%)**
**Target TH/TKA stays**	**143 227**
Target THA	75 311
Target TKA	67 916
**HCOs with at leat 1 THA/TKA target stay**	**777**
SSI detected 3 months after TH/KA replacement	1,279
HCOs with at least 1 SSI detected by ISO-ORTHO	470
**HCOs with at least 1 SSI detected in their HCO : concerned by the chart review**	**448**
**HCOs participating to the SSI chart review**	**250**
**SSI reviewed**	**725**

### Outcome

The occurrence of SSI was sought from the date of admission to 90 days after TH/KA surgery, using 15 combinations of ICD-10 codes of infection and/or complication and therapeutic or diagnostic procedure codes from the national HDD (Case definition of SSI in Table 1, and *Appendix*A.1). Any SSI occurring after 90 days has not been included.

**Table 1:** Case definition of Surgical site infections

Primary diagnosis (ICD 10 code)	Secondary diagnosis (ICD 10 code)		Procedure (procedure codes)	
**SSI detection during the TH/TKA stay**				
Infection code	Infection and inflammatory reaction due to internal joint prosthesis (T84.5)		-	
Infection code	-		Therapeutic procedure code, out of synovectomy or reoperation	
Infection code	-		Diagnostic procedure code	
-	Infection and inflammatory reaction due to internal joint prosthesis (T84.5)		Therapeutic procedure code, out of synovectomy or reoperation	
-	Infection and inflammatory reaction due to internal joint prosthesis (T84.5)		Diagnostic procedure code	
**SSI detection after discharge within 90 days following surgery**				
Infection code		Infection and inflammatory reaction due to internal joint prosthesis (T84.5)		-
Infection code		-		Therapeutic procedure code, out of synovectomy or reoperation
Infection code		-		Diagnostic procedure code
-		Infection and inflammatory reaction due to internal joint prosthesis (T84.5) + infection code		-
-		Infection and inflammatory reaction due to internal joint prosthesis (T84.5) + infection code		Therapeutic procedure code, out of synovectomy or reoperation
		Infection and inflammatory reaction due to internal joint prosthesis (T84.5) + infection code		Diagnostic procedure code
-		Infection code		Therapeutic procedure code, out of synovectomy or reoperation
Infection and inflammatory reaction due to internal joint prosthesis (T84.5) + infection code		Infection code		-
Infection and inflammatory reaction due to internal joint prosthesis (T84.5) + infection code		-		Therapeutic procedure code, out of synovectomy or reoperation
Infection and inflammatory reaction due to internal joint prosthesis (T84.5) + infection code		-		Diagnostic procedure code
-		Infection code		Diagnostic procedure code
Infection and inflammatory reaction due to internal joint prosthesis (T84.5) + infection code				Therapeutic procedure code of reintervention on TH/KA
		Infection code		Therapeutic procedure code of reintervention on TH/KA
Infection code				Therapeutic procedure code of reintervention on TH/KA
		Infection and inflammatory reaction due to internal joint prosthesis (T84.5)		Therapeutic procedure code of reintervention on TH/KA

### Assessment

The SSI detected with ISO-ORTHO were checked using a medical chart review performed in volunteer healthcare organisations (HCO) having at least one SSI detected in their patients during the 90 days following surgery, or during the TH/KA stay, or a readmission in the same HCO between January 1st and September 30th 2018. The manual review occurred between December 20th 2019 and March 24th 2020. The process of chart review was carried out on a secured platform, including access to the medical charts meeting the SSI case definition and the forms required for case analysis. The chart review was conducted under the responsibility of the medical information department. The participation of a clinician (notably a surgeon or an anaesthetist) was required. To contribute to coding quality analysis and further improvement, guidelines for SSI and TH/KA coding were also provided to the HCO [35, 36].

The assessment of PPV was conducted in two consecutive steps (i) validation of the target population to confirm the TH/KA procedure with respect to the inclusion and exclusion criteria, (ii) identification of the true positive and residual false positive SSI cases. True positive cases were defined as SSI detected by the algorithm and confirmed in the patient chart, and false positive cases as SSI detected by the algorithm and not confirmed in the patient chart. Eventually, the PPV was estimated as the percentage of true positive SSI cases divided by the total SSI cases detected by the algorithm.

## Results

Over the study period, 143,227 TH/KA stays were detected in the national HDD, coded by 777 HCO with at least one target TH/KA stay. Among them, 1,279 SSI were detected within 90 days after surgery, mostly during a readmission (97.4%)(Fig. 1). SSI rate was 0.89% in the study population, 0.98% for THA and 0.80% for TKA. The sociodemographic characteristics showed a male predominance (sex ratio 1.77), and a median age of 70 years old, independent of the prosthesis site.

Among the 777 HCO, 470 (60.5%) had at least one SSI detected within 90 days after arthroplasty. HCO reviewed patient charts of patients readmitted for SSI in the hospital where TH/KA procedure was performed, corresponding to 448 (95.3%) HCO. Of the 448 HCO, 250 HCO (56% of the 448) volunteered to perform it. A majority of them were private hospitals (68%), 22.8% were public general hospitals, and 9.2% were teaching hospitals. Their distribution of the type of HCO was similar to the 470 HCO with at least one case of SSI detected (*Appendix*A.2).

Eventually, 725 medical charts of SSI were reviewed, representing 57% of the hospital stays with SSI. 86% of the 725 medical charts were reviewed by infection control practitioners, 36% by orthopaedist surgeons, 19% by doctors specialized in medical information, 13% by doctors specialized in infectious diseases and 8.4% by anaesthesiologists.

Of the 725 reviewed SSI, 655 were confirmed SSI and 63 were not. Among the 63 cases of false positive SSI, 29 were SSI suspected but not subsequently confirmed, 10 were uninfected hematomas, 10 had missing information about a potential SSI in the medical chart, four were infections not related to the surgical site, three were related to a history of SSI, three were a revision surgery for another reason. Seventy-three target stays (10%) had at least one exclusion factor of the target population (Table I). The absence of, or incorrect, coding by HCO were the main reasons for the absence of SSI confirmation.

**Table:** Table Ibis: Review the exclusion criteria of the target population

Exclusion criteria	Target population after manual review* (N = 652)
**Surgery in the previous three months**	46 (6.3%)
**Fracture for replacement**	10 (1.4%)
**Non-French residents**	10 (1.4%)
**Transferred from another healthcare organisation**	7 (1%)
**Several acts of orthopaedic surgery**	7 (1%)
**Emergency admission**	6 (0.8%)
**Discharged against medical advice or escaped**	3 (0.4%)

* Potentially excluded for more than one criterion.

The PPV of the SSI algorithm was 90.3% [88.2%; 92.5%]. Among THA, 384 of the 427 SSI (89.9% [87.1%; 92.8%]) were true positives. Among TKA, 271 of the 298 SSI (90.9% [87.7%; 94.2%]) were true positives (Table II).

**Table:** Table II: Performance of ISO-ORTHO Algorithm overall and by surgery site

Surgery site	SSI confirmed on medical chart review	False positive SSI	True positive SSI	PPV
**THA**	427	39	384	89.9% [87.1-92.8%]
**TKA**	298	24	271	90.9% [87.7-94.2%]
**Total**	725	63	655	90.3% [88.2-92.5%]

*SSI surgical site infection; PPV predictive positive value.*

## Discussion

We successfully validated the computerized indicator ISO-ORTHO, an outcome indicator in orthopaedics assessing SSI after a TH/KA in France. We demonstrated that automated SSI detection after total hip or knee arthroplasty using mandatory and available HDD is feasible and reliable regarding its performance to detect SSI. The PPV was estimated at 90% for both the combined indicator and the individual THA or TKA measures, confirming its potential use for different purposes (e.g. quality improvement and risk management, public disclosure, financial purpose) according to the HAS method [31]. Indeed, performance parameters can vary in quality and accuracy, as previously demonstrated [37–40]. According to the coding guidelines, SSI must be coded with a dedicated ICD-10 code: T84.5 associated with an infection code. This recommended combination of codes detected 66% of the SSI confirmed by the chart review. Variation in the coding practices required the use of 15 combinations of ICD-10 and procedure codes.

ISO-ORTHO has a very high PPV compared to the PPV found in literature, reported as 63.6% and 78% in other studies [11, 40, 41]. A PPV of at least 75% [31] is essential to ensure that it is consistent with the real practice and usable for quality and safety improvements [42]. The main reasons for false positives were suspicions of SSI that were not subsequently confirmed, the presence of an uninfected hematoma and a lack of SSI occurrence in the reviewed medical charts. They are mainly linked to the incorrect use of the coding instructions for SSI regarding the clinical information in medical charts [26, 30, 36, 43]. Validation of the target population showed 10% of remaining exclusion criteria linked to the absence of traceability of the information in the medical charts and/or the absence or incorrect coding by HCO.

Our study has, however, some limitations. The use of administrative hospital databases introduced an inherent bias that should be taken into consideration. The strengths and limitations of using healthcare databases for epidemiological purposes have already been extensively discussed [27, 42, 44, 45]. The coding practices could be heterogeneous and not accurate, especially concerning the type of SSI (deep or superficial) or the joint laterality. The built algorithm selects superficial and deep SSI and takes into account only the first primary arthroplasty. Moreover, SSI are rare events. In France, the incidence estimated in 2018 based on the reports of 258 voluntary HCO was 1.35% [1.16-1.54%] for THA including hip fractures, and 0.9% [1.72%-1.08%] for TKA [43]. The SSI rates detected by ISO-ORTHO are consistent with these previous results: 0.96% for THA and 0.80% for TKA and the difference could be notably explained by the population exclusion criteria. It was chosen to control only positive predictive value for reasons of relevancy when low event rates are concerned, as well as material and human feasibility. Given the low frequency of SSI, the number of subjects required to calculate a negative predictive value would be too high. For a purpose of quality management and improvement, a manual review of the medical charts on a voluntary basis was performed,to ensure consistency [46]. This has not led to a selection bias, as the volunteer HCO were representative of the entire target HCO (types of hospitals and regions) and more than half of the detected SSI were analysed. The PPV over 75% is reliable for quality assessment purposes [31, 42, 47]. Eventually, the SSI detection was focused on the 90 days following the arthroplasty whereas the SSI definition is up to 365 days; however, previous studies showed that a majority of SSI after a primary arthroplasty occurred in the first three months [19, 48].

Another limitation of indicators based on HDD is the lack of clinical and medical device data and the absence of a dynamic link between the occurrence of complications and electronic health records [49, 50]. In the absence of exhaustive clinical registries for THA and TKA, several things would be relevant to detect SSI, including patient follow up, automated complication monitoring from clinical, microbiological and imaging data warehouses. They could also be used to assess ISO-ORTHO PPV as well as its negative predictive value and sensitivity. Indeed, the algorithms and methods developed for this study have been refined and validated by matching HDD with clinical data from hospital repositories and data from biomedical laboratories available in the clinical data warehouses [49–54].

## Conclusion

The ISO-ORTHO PPV over 90% confirms its validity to detect SSI over the 90 days following a TH/KA in France. According to the HAS method, it is suitable for healthcare quality improvement and in-hospital risk management, hospital accreditation as well as public disclosure and financial purposes. Following this validation study, 2018-19 results of the validated indicator (adjusted ratio of observed on expected SSI in the target TH/KA population) were released to the HCO in September 2020, enabling their use alongside other outcome indicators in orthopaedics using the French national HDD [31, 55–58] measuring thromboembolic events after TH/KA. They are used for in-hospital quality improvement and risk management, hospital accreditation and as decision making tools for regional and national policies; they also respond to users’ demand for more transparency and could be used for financial purposes.

## Electronic supplementary material

Below is the link to the electronic supplementary material.

Supplementary Material 1

## Data Availability

If you require more details concerning data and materials, please contact us by sending an email to: leslie.guillon@univ-tours.fr.

## Abbreviations

ICD-10: International classification of diseases, 10th version
“Haute Autorité de Santé”, HAS: French National Authority for Health
HCO: Healthcare organisations
HDD: Hospital discharge database
PPV: Positive predictive value
SSI: Surgical site infections
THA: Total hip arthroplasty
TKA: Total knee arthroplasty
