# Supplementary material for: Validation of the first computerized indicator for orthopaedic surgical site infections in France: ISO-ORTHO
Source: Antimicrob Resist Infect Control. 2023 May 4;12:44. doi: 10.1186/s13756-023-01239-7 (PMC10161661; doi:10.1186/s13756-023-01239-7)
Supplement: Supplementary file 1 — Supplementary Material 1 [file 13756_2023_1239_MOESM1_ESM.docx]

Appendix A.1: Arguments for inclusion and exclusion criteria and codes used 3

[Table 1: Diagnosis of infection 7](#_Toc99626521)

Table 2: Interventional procedures hip and knee 9

Table 3: Complication codes 14

Table 4: Therapeutic procedures codes 14

Table 5: Diagnostic procedures 15

Table 6: Revision procedures 15

[Appendix A.2: Distribution of the HCO with at least one SSI identified via the HDD algorithm. 15](#_Toc99626527)

# Appendix A.1: Arguments for inclusion and exclusion criteria of the target population and codes used for THA / TKA and SSI detection

| **ISO-ORTHO** | **Arguments** |
| --- | --- |
| **Target population** | |
| PMSI MCO stays chained discharged between 1^st^ January and 30^th^ September 2017 (at the geographic FINESS level)  **Inclusion criteria**   - Inclusion of the first stays on the study period with a surgical act of THA or TKA. - List of CCAM codes for THA surgical act: NEKA010, NEKA012, NEKA013, NEKA014, NEKA015, NEKA016, NEKA017, NEKA019, NEKA020, NEKA021 - List of CCAM codes for TKA surgical act: NFKA007, NFKA008, NFKA009   **Exclusion criteria**   - Stays with grouping errors (CMD 90 Errors and other unclassifiable stays) - Sessions (CMD 28 Sessions) - CMD 14 stays: pregnancy, childbirth and post-partum disorders and CMD 15 new-born, prematurity and perinatal disorders - ~~Inconsistent stays~~ ? - Stays of patients age under 18 - Death during the surgical stay without any code of SSI - Admission for SSI on the first stay (***Table 1***) - Stay with at least one complication code corresponding to an orthopaedic infectious complication, other than joint replacements: T84.6 or T84.7 - Stays with an ICD-10 main code of fracture: M80.x, M84.1, M84.2, M84.3, M84.4, M90.7, S32.x, S72.x, S79.x, M96.6, associated or not to traumatism codes (S00 - S99, T00-T07) - Stays with at least a ~~S~~second arthroplasty replacement during the THA/TKA ~~first placement~~ (only single ~~placements~~ procedures are kept) - Stays with at least one CCAM procedure act of removal, replacement or exchange of prosthesis, without SSI coded in the readmission: - Hip device replacement: NEKA001, NEKA003, NEKA006, NEKA008, NEKA022 (suppression NEKA002, NEKA004, NEKA005, NEKA007, NEKA009) - Hip device exchange: NELA001, NELA002, NELA003 - Hip device removal: NEGA001, NEGA002, NEGA003, NEGA004, NEGA005 - Knee device replacement: NFKA001, NFKA002, NFKA003, NFKA004, NFKA005 - Knee device exchange: NFLA001, NFLA002, NAGA001 - Knee device removal: NFGA002, NFGA001 - TK/HA stays associated with a mechanical complication (T84.0, T84.1) which reflects dislocation or fracture of existing prosthesis - Stays of patients coming from another facility through transfer, transfer or inter-facility benefit (entry code n°6, 7 or 0) - Stays of patients admitted by the emergency department (entry code n°5 or 8 or 1st step in post emergency unit) - Stays for surgery followed by at least one other stay with a CCAM procedure for hip or knee surgery between the stay for surgery and the stay for rehospitalisation for SSI (***Table 2***) - Stays of patients with a history of hospitalization for hip or knee surgery in the 3 months preceding the ~~insertion~~ THA/TKA stay (***Table 2***) - Stays for patients with a history of complex osteoarticular infection coded as DAS in the year prior to the installation stay (Z76.800) - Stays of residing outside of France (Geographic codes between 99101 and 99517 + 99999) - Patients with at least on ICD10 code of palliative care during the first stay or the follow up (Z51.5) - Length of stay over 90 days - Stays of patients discharged against medical advice or escaped (Z53.2) | Partial/intermediate hip or knee prosthesis are not included. Only total hip and knee prosthesis are considered, according to the list provided by the ATIH.  These stays correspond to infectious complication not related to the arthroplasty replacement and not attributable to the quality management. The concerned stays associated to a mechanical complication (T84.0 et T84.1) are excluded  Exclusion if association with a procedure code of hip fracture or polytrauma context  The list of exclusions for prosthesis changes and replacements has been completed by procedures for the removal of hip and knee material or prostheses coded during the stay for the insertion of a THP or a TKP  Exclusion of T codes for infection on existing material or other prosthesis (T84.6 and T84.7). This makes it possible to exclude a priori cases of infection not related to the installation of a total hip or knee prosthesis but related to an existing material on the same site or on another orthopaedic site. This also allows for the correction of possible errors in the use of PTH or PTG codes for patients who have already had a material or partial prosthesis inserted  The list of hip/knee surgery procedures targets the following locations: coxal bone, proximal femur, knee, patella and proximal tibia)  Exclusion of stays with an Z76800 code of complex SSI in the year before the first stay  Maximum length of stay is 90-days. |
| **SSI detection** | |
| SSI detected in the stays of target population:   - In the stay of arthroplasty replacement  \| Main diagnosis  (ICD-10 codes) \| Associated diagnosis  (ICD-10 codes) \| Act  (CCAM codes) \| \| --- \| --- \| --- \| \| Infection diagnosis \| Complication codes (T84.5) \| - \| \| Infection diagnosis \| - \| Therapeutic procedures codes \| \| Infection diagnosis \| - \| Therapeutic procedures codes \| \| - \| T84.5 \| Therapeutic procedures codes \| \| - \| T84.5 \| Therapeutic procedures codes \|  - In a readmission stay for SSI in the 90 days follow up (the first readmission with SSI is considered)  \| Main diagnosis  (ICD-10 codes) \| Associated diagnosis  (ICD-10 codes) \| Act (CCAM codes) \| \| --- \| --- \| --- \| \| Infection diagnosis \| T84.5 \| - \| \| Infection diagnosis \| - \| Therapeutic procedures codes \| \| Infection diagnosis \| - \| Therapeutic procedures codes \| \| - \| T84.5 &  Infection diagnosis \| - \| \| - \| T84.5 \| Therapeutic procedures codes \| \|  \| T84.5 \| Therapeutic procedures codes \| \| - \| Infection diagnosis \| Therapeutic procedures codes \| \| T84.5 \| Infection diagnosis \| - \| \| T84.5 \| - \| Therapeutic procedures codes \| \| T84.5 \| - \| Diagnosis procedures codes \| \| - \| Infection diagnosis \| Diagnosis procedures codes \| \| T84.5 \| - \| Surgical revision procedures codes \| \| - \| Infection diagnosis \| Surgical revision procedures codes \| \| Infection diagnosis \| - \| Surgical revision procedures codes \| \| - \| T84.5 \| Surgical revision procedures codes \|   Infection diagnosis: ***Table 1***  Complication codes: ***Table 3***  Therapeutic procedures codes: ***Table 4***  Diagnosis procedure codes: ***Table 5***  Surgical revision procedures codes: ***Table 6*** | SSI detection codes are targeted on osteoarticular infections (***Table 1***)  The complication code recommended by ATIH is retained to detect SSI (T84.5 - « Infection and inflammatory reaction due to an internal joint prosthesis »)  The algorithm for detecting SSI during the first stay and in the readmission stay have been distinguished:   - Refinement of the combinations to be taken into account, in particular to distinguish those with a diagnostic procedure from those with a therapeutic procedure (**Table 4 et 5**). - Synovectomy procedures were not included in the therapeutic procedures for SSI during the stay. Some institutions incorrectly code synovectomy procedures with prosthesis insertion procedures, even when there is no infectious context. - Joint exploration (arthroscopy) is not used as a diagnostic procedure, as it is not specific to the treatment of SSI on prosthesis.   Revision of TK/HA procedure codes are included to the list of therapeutic procedures (removal, insertion, replacement and replacement of part or all of a total prosthesis) for the detection of SSI during follow-up (**Table 6**). |

## Table 1: Diagnosis of infection

| **Code** | **Label** |
| --- | --- |
| **A180** | Tuberculosis of bones and joints |
| **A666** | Bone and joint lesions of yaws |
| **B453** | Cryptococcosis |
| **B672** | Echinococcus granulosus infection of bone |
| **M000** | Staphylococcal arthritis and polyarthritis |
| **M0000** | Staphylococcal polyarthritis |
| **M0005** | Staphylococcal arthritis, hip |
| **M0006** | Staphylococcal arthritis, knee |
| **M0008** | Staphylococcal arthritis, vertebrae |
| **M0009** | Staphylococcal arthritis, unspecified joint |
| **M001** | Pneumococcal arthritis and polyarthritis |
| **M0010** | Pneumococcal polyarthritis |
| **M0015** | Pneumococcal arthritis, hip |
| **M0016** | Pneumococcal arthritis, knee |
| **M0018** | Pneumococcal arthritis, vertebrae |
| **M0019** | Pneumococcal arthritis, unspecified joint |
| **M002** | Other streptococcal arthritis and polyarthritis |
| **M0020** | Other streptococcal polyarthritis |
| **M0025** | Other streptococcal arthritis, hip |
| **M0026** | Other streptococcal arthritis, knee |
| **M0028** | Other streptococcal arthritis, vertebrae |
| **M0029** | Other streptococcal arthritis, unspecified joint |
| **M008** | Arthritis and polyarthritis due to other bacteria |
| **M0080** | Polyarthritis due to other bacteria |
| **M0085** | Arthritis due to other bacteria, hip |
| **M0086** | Arthritis due to other bacteria, knee |
| **M0088** | Arthritis due to other bacteria, vertebrae |
| **M0089** | Arthritis due to other bacteria, unspecified joint |
| **M009** | Pyogenic arthritis and polyarthritis |
| **M0090** | Pyogenic polyarthritis |
| **M0095** | Pyogenic arthritis, hip |
| **M0096** | Pyogenic arthritis, knee |
| **M0098** | Pyogenic arthritis, vertebrae |
| **M0099** | Pyogenic arthritis, unspecified joint |
| **M010** | Meningococcal arthritis and polyarthritis |
| **M0100** | Meningococcal polyarthritis |
| **M0105** | Meningococcal arthritis, hip |
| **M0106** | Meningococcal arthritis, knee |
| **M0108** | Meningococcal arthritis, vertebrae |
| **M0109** | Meningococcal arthritis, unspecified joint |
| **M011** | Tuberculous arthritis |
| **M0110** | Tuberculous polyarthritis |
| **M0115** | Tuberculous arthritis, hip |
| **M0116** | Tuberculous arthritis, knee |
| **M0118** | Tuberculous arthritis, vertebrae |
| **M0119** | Tuberculous arthritis, unspecified joint |
| **M012** | Arthritis and polyarthritis in Lyme disease |
| **M0120** | Polyarthritis in Lyme disease |
| **M0125** | Arthritis in Lyme disease, hip |
| **M0126** | Arthritis in Lyme disease, knee |
| **M0128** | Arthritis in Lyme disease, vertebrae |
| **M0129** | Arthritis in Lyme disease, unspecified joint |
| **M013** | Arthritis and polyarthritis in other bacterial diseases classified elsewhere |
| **M0130** | Polyarthritis in other bacterial diseases classified elsewhere |
| **M0135** | Arthritis in other bacterial diseases classified elsewhere, hip |
| **M0136** | Arthritis in other bacterial diseases classified elsewhere, knee |
| **M0138** | Arthritis in other bacterial diseases classified elsewhere, vertebrae |
| **M0139** | Arthritis in other bacterial diseases classified elsewhere, unspecified joint |
| **M016** | Arthritis and polyarthritis in mycoses |
| **M0160** | Polyarthritis in mycoses |
| **M0165** | Arthritis in mycoses, hip |
| **M0166** | Arthritis in mycoses, knee |
| **M0168** | Arthritis in mycoses, vertebrae |
| **M0169** | Arthritis in mycoses, unspecified joint |
| **M018** | Arthritis and polyarthritis in other infectious and parasitic diseases classified elsewhere |
| **M0180** | Polyarthritis in other infectious and parasitic diseases classified elsewhere |
| **M0185** | Arthritis in other infectious and parasitic diseases classified elsewhere, hip |
| **M0186** | Arthritis in other infectious and parasitic diseases classified elsewhere, knee |
| **M0188** | Arthritis in other infectious and parasitic diseases classified elsewhere, vertebrae |
| **M0189** | Arthritis in other infectious and parasitic diseases classified elsewhere, unspecified joint |
| **M860** | Acute hematogenous osteomyelitis |
| **M8600** | Acute hematogenous osteomyelitis, multiple sites |
| **M8605** | Acute hematogenous osteomyelitis, femur |
| **M8606** | Acute hematogenous osteomyelitis, tibia and fibula |
| **M8608** | Acute hematogenous osteomyelitis, other sites |
| **M8609** | Acute hematogenous osteomyelitis, unspecified site |
| **M861** | Other acute osteomyelitis |
| **M8610** | Other acute osteomyelitis, multiple sites |
| **M8615** | Other acute osteomyelitis, femur |
| **M8616** | Other acute osteomyelitis, tibia and fibula |
| **M8618** | Other acute osteomyelitis, other sites |
| **M8619** | Other acute osteomyelitis, unspecified sites |
| **M862** | Subacute osteomyelitis |
| **M8620** | Subacute osteomyelitis, multiple sites |
| **M8625** | Subacute osteomyelitis, femur |
| **M8626** | Subacute osteomyelitis, tibia and fibula |
| **M8628** | Subacute osteomyelitis, other sites |
| **M8629** | Subacute osteomyelitis, unspecified sites |
| **M863** | Chronic multifocal osteomyelitis |
| **M8630** | Chronic multifocal osteomyelitis, multiple sites |
| **M8635** | Chronic multifocal osteomyelitis, femur |
| **M8636** | Chronic multifocal osteomyelitis, tibia and fibula |
| **M8638** | Chronic multifocal osteomyelitis, other sites |
| **M8639** | Chronic multifocal osteomyelitis, unspecified sites |
| **M864** | Chronic osteomyelitis with draining sinus |
| **M8640** | Chronic osteomyelitis with draining sinus, multiple sites |
| **M8645** | Chronic osteomyelitis with draining sinus, femur |
| **M8646** | Chronic osteomyelitis with draining sinus, tibia and fibula |
| **M8648** | Chronic osteomyelitis with draining sinus, other sites |
| **M8649** | Chronic osteomyelitis with draining sinus, unspecified sites |
| **M865** | Other chronic hematogenous osteomyelitis |
| **M8650** | Other chronic hematogenous osteomyelitis, multiple sites |
| **M8655** | Other chronic hematogenous osteomyelitis, femur |
| **M8656** | Other chronic hematogenous osteomyelitis, tibia and fibula |
| **M8658** | Other chronic hematogenous osteomyelitis, other sites |
| **M8659** | Other chronic hematogenous osteomyelitis, unspecified sites |
| **M866** | Other chronic osteomyelitis |
| **M8660** | Other chronic osteomyelitis, multiple sites |
| **M8665** | Other chronic osteomyelitis, femur |
| **M8666** | Other chronic osteomyelitis, tibia and fibula |
| **M8668** | Other chronic osteomyelitis, other sites |
| **M8669** | Other chronic osteomyelitis, unspecified sites |
| **M868** | Other osteomyelitis |
| **M8680** | Other osteomyelitis, multiple sites |
| **M8685** | Other osteomyelitis, femur |
| **M8686** | Other osteomyelitis, tibia and fibula |
| **M8688** | Other osteomyelitis, other sites |
| **M8689** | Other osteomyelitis, unspecified sites |
| **M869** | Osteomyelitis, unspecified |
| **M8690** | Osteomyelitis, unspecified, mutiple sites |
| **M8695** | Osteomyelitis, unspecified, femur |
| **M8696** | Osteomyelitis, unspecified, tibia and fibula |
| **M8698** | Osteomyelitis, unspecified, other sites |
| **M8699** | Osteomyelitis, unspecified, unspecified sites |
| **M900** | Tuberculosis of bone |
| **M9000** | Tuberculosis of bone, multiple sites |
| **M9005** | Tuberculosis of bone, femur |
| **M9006** | Tuberculosis of bone, tibia and fibula |
| **M9008** | Tuberculosis of bone, other sites |
| **M9009** | Tuberculosis of bone, unspecified sites |
| **M901** | Periostitis in other infectious diseases classified elsewhere |
| **M9010** | Periostitis in other infectious diseases classified elsewhere, multiple sites |
| **M9015** | Periostitis in other infectious diseases classified elsewhere, femur |
| **M9016** | Periostitis in other infectious diseases classified elsewhere, tibia and fibula |
| **M9018** | Periostitis in other infectious diseases classified elsewhere, other sites |
| **M9019** | Periostitis in other infectious diseases classified elsewhere, unspecified sites |
| **M902** | Osteopathy in other infectious diseases classified elsewhere |
| **M9020** | Osteopathy in other infectious diseases classified elsewhere, multiple sites |
| **M9025** | Osteopathy in other infectious diseases classified elsewhere, femur |
| **M9026** | Osteopathy in other infectious diseases classified elsewhere, tibia and fibula |
| **M9028** | Osteopathy in other infectious diseases classified elsewhere, other sites |
| **M9029** | Osteopathy in other infectious diseases classified elsewhere, unspecified sites |

## Table 2: Interventional procedures hip and knee

| **CCAM** | **Localisation** |
| --- | --- |
| **NAHA002** | Hip |
| **NAHA001** | Hip |
| **NEHA002** | Hip |
| **NEQC001** | Hip |
| **NFQC001** | Hip |
| **NAEP002** | Hip |
| **NAEP001** | Hip |
| **NACB001** | Hip |
| **NACA001** | Hip |
| **NACA002** | Hip |
| **NACA005** | Hip |
| **NACA003** | Hip |
| **NACA004** | Hip |
| **NAPA004** | Hip |
| **NAPA003** | Hip |
| **NAPA005** | Hip |
| **NAPA001** | Hip |
| **NAPA007** | Hip |
| **NAPA002** | Hip |
| **NAPA008** | Hip |
| **NAPA006** | Hip |
| **NAFA002** | Hip |
| **NAGA003** | Hip |
| **NAGA002** | Hip |
| **NAFA004** | Hip |
| **NAFA006** | Hip |
| **NAFA003** | Hip |
| **NAFA001** | Hip |
| **NAMA002** | Hip |
| **NAGA001** | Hip |
| **NBEP002** | Knee |
| **NBEP001** | Hip |
| **NBEB001** | Hip |
| **NBCA012** | Hip |
| **NBCA005** | Hip |
| **NBCA010** | Hip |
| **NBCA008** | Hip |
| **NBCA004** | Hip |
| **NBCA009** | Hip |
| **NBCA006** | Hip |
| **NBCB001** | Knee / Hip |
| **NBCB002** | Knee / Hip |
| **NBCB004** | Knee / Hip |
| **NBCA007** | Knee / Hip |
| **NBCB006** | Knee |
| **NBCA014** | Knee |
| **NBCA015** | Knee |
| **NBCA003** | Knee |
| **NBCA013** | Knee |
| **NFDC001** | Knee |
| **NFDA009** | Knee |
| **NBCB005** | Knee |
| **NBCA011** | Knee |
| **NBPA019** | Hip |
| **NBPA014** | Hip |
| **NBPA020** | Hip |
| **NBPA013** | Hip |
| **NBPA018** | Knee |
| **NBPA006** | Knee |
| **NBFA001** | Knee / Hip |
| **NBGA002** | Knee / Hip |
| **NBGA006** | Knee / Hip |
| **NBGA003** | Hip |
| **NBGA005** | Hip |
| **NBGA004** | Knee |
| **NBGA001** | Knee |
| **NBFA003** | Knee / Hip |
| **NBFA004** | Hip |
| **NBFA008** | Hip |
| **NBFA002** | Knee / Hip |
| **NBFA006** | Knee / Hip |
| **NBFA007** | Knee / Hip |
| **NBMA003** | Knee / Hip |
| **NBMA001** | Knee / Hip |
| **NBDA004** | Knee |
| **NBDA013** | Knee |
| **NBGA015** | Knee |
| **NBGA010** | Knee |
| **NBGA007** | Hip |
| **NBPA016** | Hip |
| **NBPA005** | Hip |
| **NBCB003** | Hip |
| **NBCA002** | Knee |
| **NBCA001** | Knee |
| **NBFA005** | Knee |
| **NBFA009** | Knee |
| **NBMA002** | Knee |
| **NEJB001** | Hip |
| **NEJA002** | Hip |
| **NEJA004** | Hip |
| **NEEP006** | Hip |
| **NEEP007** | Hip |
| **NEEP004** | Hip |
| **NEEP005** | Hip |
| **NEEP002** | Hip |
| **NEEA004** | Hip |
| **NEEA001** | Hip |
| **NEEA003** | Hip |
| **NEEA002** | Hip |
| **NEJC001** | Hip |
| **NEJA001** | Hip |
| **NEDA001** | Hip |
| **NEMA021** | Hip |
| **NEMA003** | Hip |
| **NEMA017** | Hip |
| **NEDA002** | Hip |
| **NEMA020** | Hip |
| **NEMA018** | Hip |
| **NEKA018** | Hip |
| **NEKA011** | Hip |
| **NELA003** | Hip |
| **NEKA020** | Hip |
| **NEKA012** | Hip |
| **NEKA014** | Hip |
| **NEKA010** | Hip |
| **NEKA016** | Hip |
| **NEKA017** | Hip |
| **NEKA021** | Hip |
| **NEKA015** | Hip |
| **NEKA013** | Hip |
| **NEKA019** | Hip |
| **NEGA004** | Hip |
| **NEGA005** | Hip |
| **NEGA002** | Hip |
| **NEGA003** | Hip |
| **NEGA001** | Hip |
| **NEKA022** | Hip |
| **NEKA004** | Hip |
| **NEKA009** | Hip |
| **NEKA002** | Hip |
| **NEKA007** | Hip |
| **NEKA005** | Hip |
| **NEKA003** | Hip |
| **NEKA008** | Hip |
| **NEKA006** | Hip |
| **NEKA001** | Hip |
| **NELA002** | Hip |
| **NELA001** | Hip |
| **NEFA001** | Hip |
| **NEMA013** | Hip |
| **NEMA011** | Hip |
| **NEPA001** | Hip |
| **NEFC001** | Hip |
| **NEFA004** | Hip |
| **NEFA003** | Hip |
| **NFJC002** | Knee |
| **NFJA002** | Knee |
| **NFEP002** | Knee |
| **NFJC001** | Knee |
| **NFJA001** | Knee |
| **NFDA002** | Knee |
| **NFDA003** | Knee |
| **NFKA009** | Knee |
| **NFKA006** | Knee |
| **NFKA007** | Knee |
| **NFKA008** | Knee |
| **NFGA002** | Knee |
| **NFGA001** | Knee |
| **NFKA004** | Knee |
| **NFKA003** | Knee |
| **NFKA005** | Knee |
| **NFKA001** | Knee |
| **NFKA002** | Knee |
| **NFLA002** | Knee |
| **NFLA001** | Knee |
| **NFMA013** | Knee |
| **NFMA006** | Knee |
| **NFPC002** | Knee |
| **NFPA001** | Knee |
| **NFPA003** | Knee |
| **NFFC002** | Knee |
| **NFFA004** | Knee |
| **NFFA005** | Knee |
| **NFFC001** | Knee |
| **NFFA002** | Knee |
| **NFFA006** | Knee |
| **NFCC002** | Knee |
| **NFCA001** | Knee |
| **NFCA004** | Knee |
| **NFCC001** | Knee |
| **NFCA006** | Knee |
| **NFCA005** | Knee |
| **NFCA003** | Knee |
| **NFMC003** | Knee |
| **NFMA004** | Knee |
| **NFMC002** | Knee |
| **NFMA010** | Knee |
| **NFMC005** | Knee |
| **NFMA008** | Knee |
| **NFMC001** | Knee |
| **NFMA011** | Knee |
| **NFCA002** | Knee |
| **NFMA005** | Knee |
| **NFMA002** | Knee |
| **NFPC001** | Knee |
| **NFPA002** | Knee |
| **NFPA004** | Knee |
| **NFFC004** | Knee |
| **NFFA003** | Knee |
| **NFFC003** | Knee |
| **NFFA001** | Knee |
| **NFEC002** | Knee |
| **NFEA002** | Knee |
| **NFEC001** | Knee |
| **NFEA001** | Knee |

## Table 3: Complication codes

| **CIM-10** | **Libellé** |
| --- | --- |
| **T84.5** | Infection and inflammatory reaction due to unspecified internal joint |
| **T84.6** | Infection and inflammatory reaction due to internal fixation device of unspecified site |
| **T84.7** | Infection and inflammatory reaction due to other internal orthopaedic prosthetic devices, implants and grafts |
| **Z76.800** | Persons encountering health services in other specified circumstances |

##

## Table 4: Therapeutic procedures codes

| **CCAM** | **Localisation** | **ISO-ORTHO (TH/TKA stay)** | **ISO-ORTHO (readmission within 90 days following surgery)** |
| --- | --- | --- | --- |
| **NEJA001** | Hip | X | X |
| **NEJA002** | Hip | X | X |
| **NEJA003** | Hip | X | X |
| **NEJA004** | Hip | X | X |
| **NEJC001** | Hip | X | X |
| **NEJB001** | Hip | X | X |
| **NEQC001** | Hip | - | - |
| **NFJA001** | Knee | X | X |
| **NFJA002** | Knee | X | X |
| **NFJC001** | Knee | X | X |
| **NFJC002** | Knee | X | X |
| **NZJB001** | Knee/Hip | X | X |
| **NEFC001** | Hip | - | X |
| **NEFA003** | Hip | - | X |
| **NEFA004** | Hip | - | X |
| **NFFC001** | Knee | - | X |
| **NFFC002** | Knee | - | X |
| **NFFA002** | Knee | - | X |
| **NFFA004** | Knee | - | X |
| **NFFA005** | Knee | - | X |
| **NFFA006** | Knee | - | X |
| **NFQC001** | Knee | - | - |

## Table 5: Diagnostic procedures

| **CCAM** | **Localisation** |
| --- | --- |
| **NAHB001** | Hip |
| **NAHA001** | Hip |
| **NAHA002** | Hip |
| **NEHA001** | Hip |
| **NEHA002** | Hip |
| **NZHA001** | Knee/Hip |
| **NZHB001** | Knee/Hip |
| **NZHB002** | Knee/Hip |
| **NZHH001** | Knee/Hip |
| **NZHH002** | Knee/Hip |
| **NZHH003** | Knee/Hip |
| **NZHH004** | Knee/Hip |

## Table 6: Revision procedures

| **CCAM** | **Localisation** |
| --- | --- |
| **NEGA004** | Hip |
| **NEGA002** | Hip |
| **NEGA003** | Hip |
| **NEGA001** | Hip |
| **NEKA004** | Hip |
| **NEKA009** | Hip |
| **NEKA002** | Hip |
| **NEKA007** | Hip |
| **NEKA005** | Hip |
| **NEKA003** | Hip |
| **NEKA008** | Hip |
| **NEKA006** | Hip |
| **NEKA001** | Hip |
| **NELA002** | Hip |
| **NELA001** | Hip |
| **NFKA004** | Knee |
| **NFKA001** | Knee |
| **NFKA002** | Knee |

# Appendix A.2: Distribution of the HCO with at least one detected SSI.

HCO Healthcare organization; SSI surgical site infection; HDD hospital discharge database.

|  | **HCO concerned by the algorithm**  **(N = 777)** | **HCO with at least one SSI case detected**  **(N = 470)** | **Participating HCO**  **(N = 250)** |
| --- | --- | --- | --- |
| **Public general Hospitals (%)** | 280 (36%) | 135 (28.7%) | 57 (22.8%) |
| **Academic Hospitals (%)** | 61 (7.9%) | 43 (9.2%) | 23 (9.2%) |
| **Private hospitals (%)** | 436 (56.1%) | 292 (62.1%) | 170 (68%) |
